# Determination of Optimum Machining Parameters for Face Milling Process of Ti6A14V Metal Matrix Composite

**DOI:** 10.3390/ma15144765

**Published:** 2022-07-07

**Authors:** Layatitdev Das, Rakesh Nayak, Kuldeep K. Saxena, Jajneswar Nanda, Shakti Prasad Jena, Ajit Behera, Shankar Sehgal, Chander Prakash, Saurav Dixit, Dalael Saad Abdul-Zahra

**Affiliations:** 1 Department of Mechanical Engineering, VSSUT Burla, Burla 768018, India; das.layatitdev_mech@vssut.ac.in (L.D.); rakesnayak123@gmail.com (R.N.); 2 Department of Mechanical Engineering, GLA University, Mathura 281406, India; saxena0081@gmail.com; 3 Department of Mechanical Engineering, ITER, SOA University, Bhubaneswar 751030, India; jajneswarnanda@soa.ac.in; 4 Department of Mechanical Engineering, NIT Bhubaneswar, Bhubaneswar 754005, India; jenashaktip@gmail.com; 5 Department of Metallurgical & Materials Engineering, NIT Rourkela, Rourkela 769008, India; 6 Mechanical Engineering, U.I.E.T., Sector-25, Panjab University, Chandigarh 160014, India; sehgals@pu.ac.in; 7 School of Mechanical Engineering, Lovely Professional University, Phagwara 144001, India; 8 Division of Research and Development, Lovely Professional University, Phagwara 144001, India; 9 Peter the Great St. Petersburg Polytechnic University, Saint Petersburg 195251, Russia; 10 Division of Research & Innovation, Uttaranchal University, Dehradun 248007, India; 11 Department of Medical Physics, Hilla University College, Babylon 51001, Iraq; dalael20175@gmail.com

**Keywords:** Ti6Al4V, facing operation, Taguchi method, grey relational analysis, ANOVA, optimization

## Abstract

This paper shows the novel approach of Taguchi-Based Grey Relational Analysis of Ti6Al4V Machining parameter. Ti6Al4V metal matrix composite has been fabricated using the powder metallurgy route. Here, all the components of TI6Al4V machining forces, including longitudinal force (F_x_), radial force (F_y_), tangential force (F_z_), surface roughness and material removal rate (MRR) are measured during the facing operation. The effect of three process parameters, cutting speed, tool feed and cutting depth, is being studied on the matching responses. Orthogonal design of experiment (Taguchi L9) has been adopted to execute the process parameters in each level. To validate the process output parameters, the Grey Relational Analysis (GRA) optimization approach was applied. The percentage contribution of machining parameters to the parameter of response performance was interpreted through variance analysis (ANOVA). Through the GRA process, the emphasis was on the fact that for TI6Al4V metal matrix composite among all machining parameters, tool feed serves as the highest contribution to the output responses accompanied by the cutting depth with the cutting speed in addition. From optimal testing, it is found that for minimization of machining forces, maximization of MRR and minimization of Ra, the best combinations of input parameters are the 2nd stage of cutting speed (175 m/min), the 3rd stage of feed (0.25 mm/edge) as well as the 2nd stage of cutting depth (1.2 mm). It is also found that hardness of Ti6Al4V MMC is 59.4 HRA and composition of that material remain the same after milling operation.

## 1. Introduction

A metal matrix composite system is generally designed by the metal alloy with the combination of the matrix and the material type, volume fraction, and form of the reinforcement. It is proved that pure titanium can be strengthened by adding aluminum for α-phase and vanadium for β-phase. The presence of α + β phase in the Ti-6Al-4V alloy increases its mechanical strength [1,2,3,4]. In addition, aluminum is lightweight, and vanadium is a high strength material, which fevers in many high-end applications. Now-a-days, titanium alloys are used in aerospace, medical science and automobiles because it has a low weight-to-strength ratio, high corrosive resistance and low thermal conductivity. In the field of medical science, Ti alloys are used for making implants and surgical instruments. Ti alloys are also widely used in aerospace engineering for the making of missiles, bearings and engines, etc. Several researchers have tried to reveal various mechanical properties of Ti-6Al-4V alloy to explore their emerging application. Nourbakhsh. et al. [5] have investigated the effect of seven different parameters including pulse width, servo reference voltage, pulse current, wire tension, cutting speed, wire rupture and surface integrity of Wire Electro-Discharge Machining (WEDM) of Titanium alloy. They found that cutting speed increases with peak current and pulse interval. The surface roughness was found to increase with pulse width and decrease with pulse interval. Yang et al. [6] reported the application of various coolant conditions, such asdry, flood coolant and minimum quantity lubrication (MQL) for processing advance materials such as Ti-6Al-4V alloys. In their work, the cutting speed was constant, while for machining uncoated and titanium aluminum nitrate (TiAlN), coated carbide tools were taken and they found various dominant properties in Ti-6Al-4V alloy.

Sahu et al. [7] have studied the effect of machining process parameters such as pulse-on time, discharge current and duty cycle on performance parameters such as material removal rate (MRR), tool wear rate (TWR) and radial over cut (ROC). They have optimized the input parameters by an optimization method named Grey Relational Analysis (GRA) and found that duty cycle has maximum percentage contribution on the output responses followed by discharge current and pulse-on time. Shokrani et al. [8] investigated the effects of cryogenic machining on surface integrity in CNC end milling of Ti-6Al-4V titanium alloy. The machining of titanium is characterized by poor surface integrity, which leads to the biggest challenge in machining operation of titanium. In their work, surface roughness and microscopic surface integrity were investigated, and surface micro hardness was measured. Their analysis indicated that cryogenic cooling results in up to 39% and 31% lower surface roughness when compared to dry and flood cooling methods, respectively. Chenthilijagan et al. [9] have successfully applied Grey Relational Analysis (GRA) for optimizing the machining parameters such as MRR, and Surface Roughness (SR). The GRA is applied to find the Grey Grade and is used to represent the multi-objective model. They concluded that GRA is the best optimization method to find the optimal solution. D’Mello. et al. [10] found that machining of Ti alloy is difficult in traditional machining because of its hardness and high heat generation whereas CNC Milling was preferred to non-conventional machining to overcome these difficulties. During machining, it was found that forces required to machine the Ti are more because of its higher hardness. These parameters are affected by cutting velocity (V), feed (F) and depth of cut (D). It was concluded that in order to produce a product with minimizing forces, maximizing MRR and minimizing the surface roughness, the re-optimization of input parameters (V, F, D) is necessary.

In recent years, the Taguchi technique has become the most widely established optimization methodology for better productivity. However, it was found by most researchers that the Taguchi technique can only optimize the single response [11,12,13]. For the optimization of more than a single response, many researchers have used a combination of Taguchi and other techniques. Ghag. et al. [14] found that the combination of the Taguchi and GRA techniques gives a close-to-the-target, economical and efficient parameter set up. Ortop et al. [15] presented a study based on a comparison between computer numeric controlled (CNC)-milled frameworks with conventional castings and evaluated the distortion caused by the use of different veneering materials. They showed that CNC frameworks had a statistically improved fit and manufacturing accuracy (*p* < 0.05) whereas veneering material had no statistically significant effect on the fit of the titanium frameworks (*p* > 0.05). The influence of electrical discharge machining (EDM) parameters such as pulse-on time (TON), pulse-off time (TOFF), voltage (V) and current (I) on material removal rate (MRR) in 304 stainless steel was explored by Rajmohan et al. [16]. The tests are carried out utilizing a L9 orthogonal array according to the design of experiments technique. The data were examined using analysis of variance and response graphs. The computational methodologies for five different approaches standardized by Gauri et al [17]. Out of the five, three sets of experimental data are examined and the anticipated optimization performances of the five methods are compared. They reveal that no approach can provide greater optimization than the weighted signal-to-noise ratio (WSN) method EDM parameters in AISI202 stainless steel by means of grey relational analysis, again presented by Chenthil et al. [9]. Their research compares the hybrid technique with Taguchi analysis. The confirmation test validates the outcome of the suggested hybrid Grey-Taguchi analysis. John et al. [18] examined the finite element analysis of the burnishing process on D3 tool steel material using a CNC lathe. The input parameters that they included were speed, burnishing force, and feed. Surface roughness, residual stress, micro-hardness and out of roundness are the output parameters. The surface roughness obtained after the turning operation is utilized to model the surface roughness pattern, which is then used to simulate the ball burnishing process using the finite element-based programme DEFORM-2D. Khare et al. [19] study the roughness on AISI 4340 steel by using the cutting speed, feed rate, depth of cut and rake angle adjusted for the lowest surface. They designed the experiment utilizing Taguchi’s L9 theory: the signal-to-noise (S/N) ratio and the Qualitek-4. Manivel et al. [20] presented the turning of ADI with carbide by varying cutting parameters. They used the Taguchi technique in this study. The cutting insert was CVD coated with AL_2_O_3_/MT TICN. The ANOVA and signal-to-noise ratio are used to identify the optimized condition to improve the cutting speed, which has a great impact on surface roughness and tool wear. Khorasani et al. [21] used selective laser melting to fabricate Ti-6Al-4V prosthetic acetabular shells. Samples were printed using the Taguchi L32 design of experiment (DOE), and multilayer perceptron (MLP) artificial neural networks were used to predict surface roughness (ANNs).

In this paper, a cylindrical sample Ti6Al4V is prepared by powder metallurgy technique, followed by CNC milling (Facing). The novelty of this research is using Taguchi-based grey relational analysis to determine the optimum machining parameter for facing operation of Ti6Al4V metal matrix composite.

## 2. Materials and Methods

**Figure 1:**
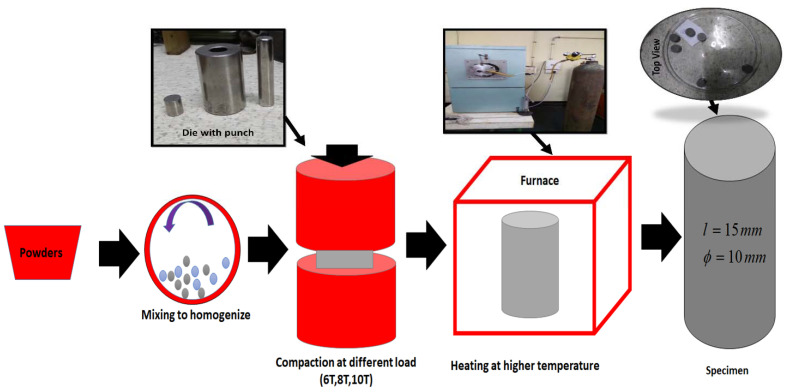
Schematics of specimen preparation.

The homogenized powder feedstock of Ti-6Al-4V alloy was prepared using planetary ball milling. Milling was carried out with a rotation speed of 50 rpm for 8 h. Then, the homogenized powder was cold-pressed into cylindrical compact with different loads. Six cylindrical samples with dimensions of 15 mm length and 10 mm diameter are prepared. From those samples, four samples are made by application of 4000 Kgf (39,226.6 N), 6000 Kgf (58,839.9 N) and 10,000 Kgf (98,066.5 N) of compact force (as shown in Table 1). Figure 1 describes the specimen preparation and dimensions of the test specimens. The die and punch used in this research work is made up of tool steel and the clearance is limited to less than 0.0254 mm. In the cold-compression process, the pressed-out are produced as green compact. The strength of these demands compatibility between the powder materials. Then, the pressed-out specimens are sintered in the presence of argon inert gas, using the Electric furnace. During this process, the temperature is increased at a rate of 40 °C per minute until the temperature of the sample reached 100 °C. Then, the temperature is maintained at 100 °C to avoid thermal cracking. Then, the temperature is increased at a rate of 50 °C per minute until the sample reaches a temperature of 1000 °C for 2 h. This isfollowed by cooling to room temperature at a rate of 50 °C per minute inside the furnace. This process is carried out in order to avoid the development of stress in the sample. The various loads required for preparation of the sample are tabulated in Table 1. The values of weight percentage of different elements present in the composition are shown in Table 2.

**Figure 2:**
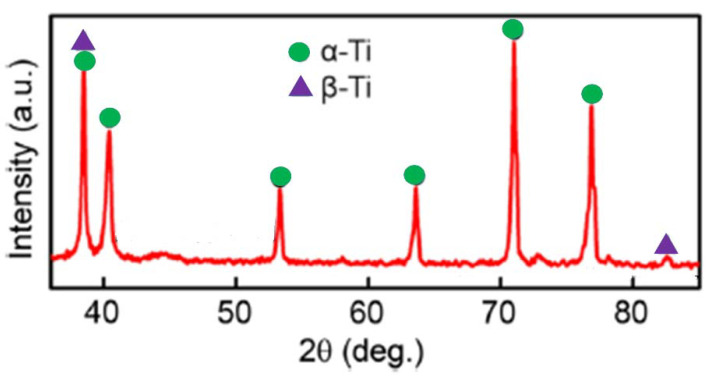
X-ray Diffraction of Ti-6Al-4V MMC.

Machining by CNC facing operation (using Ace Micrometric-MCV-450) has been carried out for all the samples. It’s a powerful and high-performance machine suitable for various applications, loaded with a high-rigidy and high-speed spindle. In this research work, an end milling operation was carried out using this milling machine. The face end milling operation is the most versatile milling operation. The end milling operation can be used to machine various slots, complex profiles and contours. In this research work, the end milling was carried out using both the end and sides of the cutter. Here, the depth of cut was given outside and then run on the body surface of the work piece. During the operation, cutting was carried out perpendicular to the work piece. The milling cutting is essentially positioned face down towards the top of the work piece. As compared to the high-speed steel cutter, the carbide cutting tool is tougher. The cutting efficiency of these tools can be maintained at high temperature. As compared to the HSS cutter, the carbide mill cutter can perform 4–8 times faster. It has high stiffness, which leads to greater resistance against wear but these cutting tools are weak and prone to cracking and peeling. In the research work, a carbide cutting tool is used in machining operation performed by the authors with a 16 mm tool diameter, 10.5 mm depth, 4 blades, a 3–4° rake angle for peripheral cutting edge with helix angle of 27°, a 0° face cutting edge, a 4° clearance angle and a 4° cutting edge inclination angle. The microstructures of the machined samples were characterized using scanning electron microscopy (JEOL JSM-6480LV) and phase analysis (Figure 2) has been carried out using X-ray diffraction (RIGAKU D/MAX-2400 diffractometer) to ensure the presence of required phases in Ti-6Al-4V metal matrix composite. The source used in XRD is Co Kα radiation (λ = 1.7909 Å), 2 mm beam diameter and the parameters used are 2θ (20° to 100°), step size (0.02) with scan rate (10°/min). Mechanical properties were investigated through a tensile test at room temperature, and all specimens for the tensile test were in their respective peak-aged conditions according to the results of age-hardening investigation. All the physical and mechanical properties are specified in the Table 3. The surface roughness of the sample has been measured using 3D surface profilometry (Dektak 150, Veeco Instruments, USA), equipped with the silicon nitride stylus tip. The mean value of roughness was calculated from the results of five successive measurements.

To identify the required forces for the removal of the materials from the TiAlV metal matrix composite, it is essential to know the optimized conditions of the machining. To optimize the machining parameters, in this work, an optimizing tool GRA combined with Taguchi has been used. GRA is a method for identifying the key features of a system by computing the Grey Relation grades. These grades are numerical measures of the impact of influencing parameters on the target variables. The larger the Grey Relation Grades the more significant impact the input has on target variables [22,23]. The advantages of GRA are its comparative simplicity and its ability to deal with a small set of data (here Taguchi L9 set of data are used) that do not have typical probability distribution. A combination of the Taguchi and GRA techniques has been used to obtain the optimal machining parameter setup in this work.

## 3. Results and Discussion 

### 3.1. Phase Analysis and Surface Morphology

Figure 2 shows the XRD pattern of the Ti-6Al-4V sample. Here, the presence of both the phase α and β phase are confirmed. Presence of α-phase is due to the introduction of Aluminum and presence of β-phase is due to the introduction of Vanadium [24,25].

**Figure 3:**
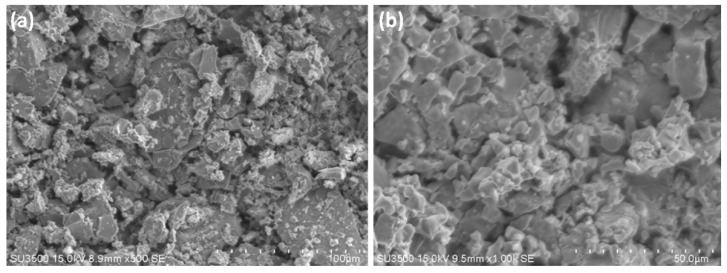
Surface morphology of the (**a**) MMC and (**b**) the fracture surface MMC.

Figure 3a depicts the surface morphology of the metal matrix composite. The dense structure of the composite at 500× magnification has been observed. Figure 3b shows the surface of the machined specimen. Smooth cutting of the composite and the presences of voids on the machined surface has been observed here.

### 3.2. Facing Operation

**Figure 4:**
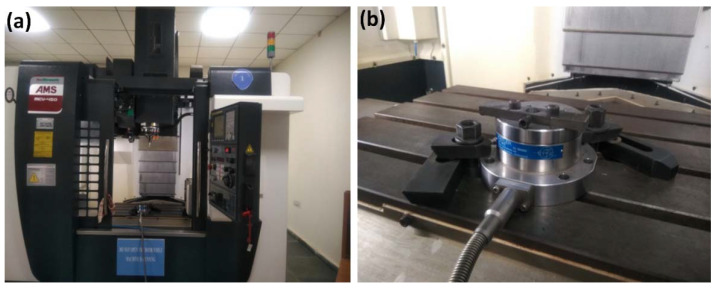
(**a**) CNC milling machine, (**b**) Tool Dynamometer in CNC milling m/c.

**Figure 5:**
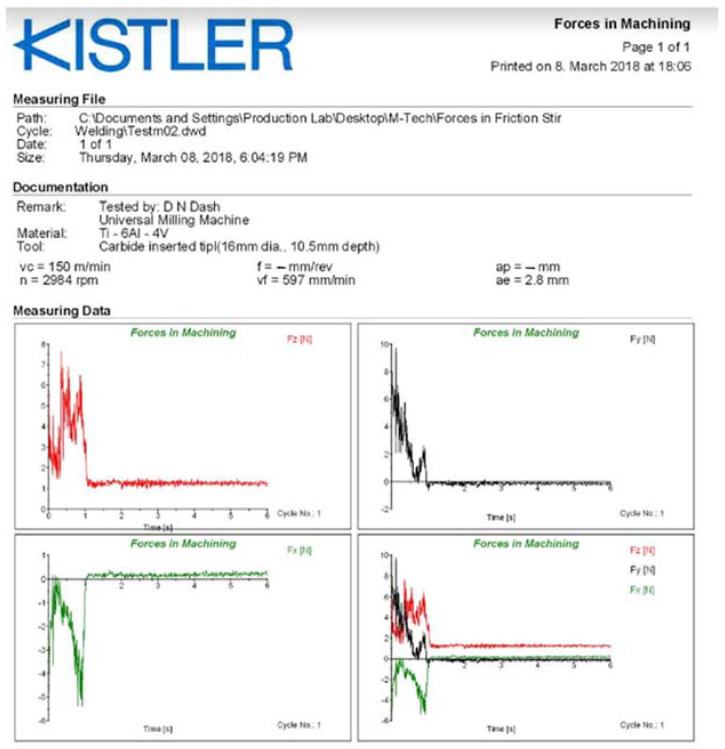
Reading obtained using tool dynamometer for various components of forces.

The facing operation is executed by CNC milling model Ace Micrometric-MCV-450 as shown in Figure 4a. The arrangement of Tool Dynamometer in CNC milling was used to record the forces experience in three mutually perpendicular directions as shown in Figure 4b. The dynamometer is used to determine the machining forces applied to the test specimens. All of the three mutually perpendicular forces, Longitudinal force (F_x_), Radial force (F_y_), Tangential force (F_z_), are displayed on dynoware software and are tabulated in Table 4. These forces are recorded by the Tool Dynamometer, which is shown in Figure 5 for all the X-, Y-, Z-component of forces.

### 3.3. Design of Experiment to Determine Responses

Taguchi L9 Orthogonal Array is used for Design of Experiment because it provides fewer runs for the experiment [26,27,28,29,30]. Cutting velocity (V), feed (F) and depth of cut (D) are taken as the input machining parameters. Machining forces [Longitudinal Force (F_x_), Radial Force (F_y_) and Tangential Force (F_z_)], MRR and Surface Roughness are calculated as output parameters [27]. Nine experiments are conducted by varying the input machining parameters with three levels of each input. *MRR* is calculated by using Equation (1). The cubic millimeter volume of material removed in one minute is known as MRR. It can be calculated per the following formula and is presented in Table 5.

(1) $$MRR=\frac{M_{f}-M_{i}}{\rho t}\;\;$$

where,

*M_f_* = Initial mass of the sample before milling
*M_i_* = Final mass of the sample after milling
*ρ* = Density of Ti alloy material
*t* = Milling time for the sample

The surface roughness, Ra is calculated using diamond tip stylus equipment. Five experiments are conducted for one sample to get better accuracy. The average of the five experimented values will give the Ra for each machined sample. Before calculating the Ra, the surfaces of the machined specimens are cleaned to remove dust. Tip of the equipment is placed on the test specimen, allow moving on it. The data are stored in the memory and are analyzed by TALUSURF software [31,32,33,34,35]. The data obtained are tabulated in Table 5.

### 3.4. Method of Optimization

The main goal of the manufacturing company is to maximize productivity by minimizing cost and improving quality. For attaining such goal, the best technique to apply in the manufacturing process is optimization. It is the method of determining the best outputs or responses by feeding input parameters. This maximizes the required outputs by minimizing input parameters [36,37,38,39,40]. There are three different types of rules used for finding quality loss ($Q_{ij}$) in the Taguchi technique: larger is best, smaller is best and nominal is best [41,42]. The number of experimental trials is e and, for each study, the performance losses of a group of performance parameters are calculated. Performance loss (*Q_ij_*) for *j*th signal with respect to *i*th output (*i* = 1, 2, ......, e; *j* = 1, 2, ......, r) for various types of response parameters are provided below Equation (2) to Equation (7) along with Table 6 and Table 7.

For smaller is the best,

(2) $$Q_{ij}=\frac{1}{n}\sum_{k=1}^{n}z_{\mathrm{ijk}}^{2}$$

For larger is the best,

(3) $$Q_{ij}=\frac{1}{n}\sum_{k=1}^{n}\frac{1}{z_{\mathrm{ijk}}^{2}}$$

For nominal is the best,

(4) $$Q_{ij}=\frac{t_{\mathrm{ij}}^{2}}{{\bar{z}}_{\mathrm{ij}}^{2}}\;$$

where, ${\bar{z}}_{\mathrm{ij}}=\frac{1}{n}\sum_{k=1}^{n}z_{\mathrm{ijk}}$, $t_{\mathrm{ij}}^{2}=\frac{1}{n\;-1}\sum_{k=1}^{n}{\left(z_{\mathrm{ijk}}-{\bar{z}}_{\mathrm{ijk}}\right)}^{2}$.

In the above Equation ‘n’ denotes the number of replication experimentations, *Z_ijk_* is the calculated values of *j*th output in *i*th inputs at *k*th replication, and *Q_ij_* is the degraded performance loss for *j*th output in *i*th inputs. The S/N value (signal-to-noise ratio) S_ij_ (Table 7) is achieved by using the value of *Q_ij_* (Table 6) for the *j*th output in the *i*th inputs by using Equation (5).

(5) $$S_{ij}=-10\log Q_{ij}\;$$

#### 3.4.1. Grey Relational Analysis Method (GRA)

In this stage of the process, the Grey Relational Grade (GRG) result is being used as the Process Performance Index (PPI). The rate of achievement of the PPI is as quantified. The value of signal to noise ratio ($S_{ij}$) is determined for output for each input by using the Equation (6),

(6) $$N_{ij}=\frac{\left(S_{ij\;}-S_{ij}^{min}\right)}{\left(S_{ij}^{max}-S_{ij}^{min}\right)}\;$$

where, $N_{ij}$ = scaled or normalized signal-to-noise ratio (Table 8) for the *j*th output in the *i*th input, $S_{ij}^{min}$ = *min* {$S_{1j}S_{2j}$, $S_{3j},\;\ldots \ldots ,\;S_{ej}$} and $S_{ij}^{max}$ = *max*{$S_{1j}S_{2j}$, $S_{3j},\;\ldots \ldots ,\;S_{ej}$}.

Gaining the S/N ratio ($S_{ij}$) values for each output for each input using Equation (6) has been given in Table 8.

#### 3.4.2. Grey Relational Coefficients Are Determined

Grey Relational coefficient ($Y_{ij}$) for *j*th output in the *i*th input is determined by using Equation (7),

(7) $$(Y_{ij})\;=\frac{\delta_{jmin}+\varepsilon \delta_{jmax}}{\delta_{ij}+\varepsilon \delta_{jmax}}\;$$

where $\delta_{ij}$ = |1 − $N_{ij}$*|*, $\delta_{jmin}$= *min* {$\delta_{1j},\;\delta_{2j},\;\ldots \ldots ,\;\delta_{ej}$}, $\delta_{jmax}$= *max* {$\delta_{1j},\;\delta_{2j},\;\ldots \ldots ,\;\delta_{ej}$}, and *ε* = distinguishing coefficient (*ε* ∊ [0, 1]). Using of the distinguishing coefficient (*ε*) is to increase or decrease the distribution of the grey relational cofficient and is often taken as 0.5 [9,23]. Determining the Grey Relationship Grade (*GRG_i_*) that communicates to the Grey Relationship Grade corresponding to the input as shown below.

(8) $$\left(GRG_{\;i}\right)=\sum_{j=1}^{p}w_{j}Y_{ij}\;$$

where $w_{j}$ is the weight for the *j*th input and $\sum_{j=1}^{p}w_{j}$ = 1.

**Figure 6:**
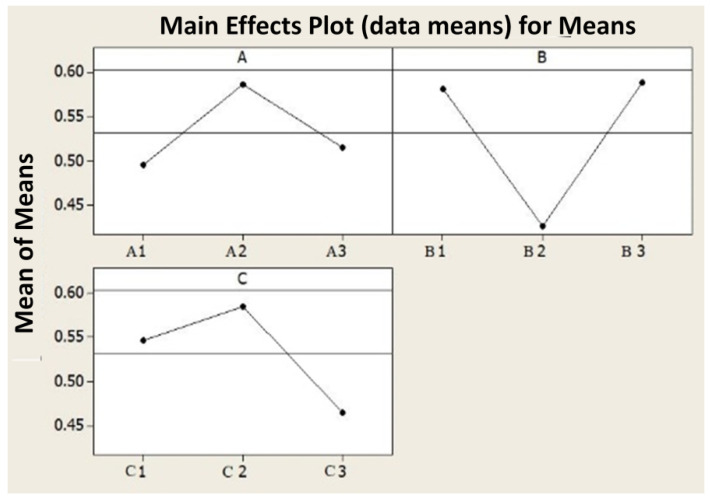
Graph for level mean of GRG values.

In this technique, the value of GRG is perceived as the value of performance index. The GRG value is calculated using Equation (8) and is shown in Table 9. The optimum value of GRG will give the maximum value of the input. The predicted optimal setting that maximizes all responses successively is the parametric combinations with the maximum GRG value. The mean level of the GRG value at each stage of the process parameters is determined and summarized in the response table (Table 10). Control variables are rated in GRG values as per their ranges. Control factors with a wide range of GRG values among their ranges have the most substantial impact on the performance. It is evident from the response table that the Tool Feed (B) has a significant impact on the output of the machining process supported by the Depth of Cut (D) and Cutting Velocity (V) factor. The response variables are shown in Figure 6.

**Figure 7:**
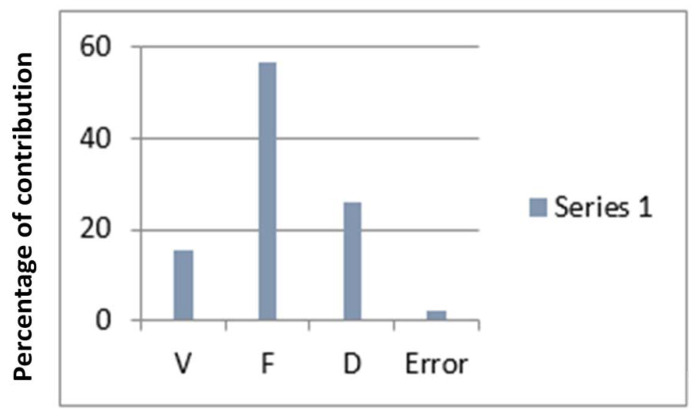
Graph for percentage of contribution of inputs for GRA technique.

Figure 7 concludes that the levels of the specific process parameters that eventually lead to the largest GRG are the 2nd stage of cutting speed (175 m/min), the 3rd stage of feed (0.25 mm/edge) as well as the 2nd stage of cutting depth (1.2 mm). The variance analysis (ANOVA) is executed on the GRG values and the results are included in Table 11. It is verified that Tool Feed (F) is the predominant control parameter accompanied by Depth of Cut (D) but mostly Cutting Velocity (V) as the former contributes a higher percentage to the overall variance. Figure 7 shows a significant contribution of all input variables, which defines the aforementioned reason.

## 4. Conclusions

This study shows the development of Ti6Al4V metal matrix composite by the powder metallurgy technique and the effect of input machining parameters (cutting speed, tool feed and cutting depth) on machining forces, MRR, surface roughness during milling. The optimization of GRA accompanied by Taguchi design of experiment is implemented for getting the optimal value of input parameters. Surface morphology and phase analysis are conducted to investigate the presence of voids and phases of the material. It is concluded from the response plot that tool feed has the highest rate contribution toward the producing responses ensued by the depth of cut and cutting velocity. From the optimal test, it is found that for minimization of machining forces, maximization of MRR and minimization of Ra, the best combinations of input parameters are the 2nd stage of cutting speed (175 m/min), the 3rd stage of feed (0.25 mm/edge) as well as the 2nd stage of cutting depth (1.2 mm). It is also found that the hardness of Ti6Al4V MMC is 59.4 HRA and the composition of the material remains thesame after milling operation.

## Figures and Tables

**Table 1:** Sample preparation using different loads.

Load Variations in Kgf (N)	Dimension of Sample		No. of Sample Made
	Length in mm	Diameter in mm	
4000 Kgf (39,226.6 N)	15	10	1
6000 Kgf (58,839.9 N)	15	10	4
10,000 Kgf (98,066.5 N)	15	10	1

**Table 2:** Composition in wt. %.

Elements	Ti	Al	V	Fe	O	C	N	H
Weight Percentage	Bal.	6.75	4.50	0.30	0.20	0.08	0.05	0.015

**Table 3:** Mechanical properties of prepared Ti-6Al-4V MMC.

	Density	Young’s Modulus (GPa)	Shear Modulus (GPa)	Bulk Modulus (GPa)	Poisson’s Ratio	Yield Strength MPa (Tensile)	Ultimate Strength MPa (Tensile)	Uniform Elongation %
Min	4.429	104	40	96.8	0.31	880	900	5
Max	4.512	113	45	153	0.37	920	950	18

**Table 4:** Input-machining parameters with their levels.

Input Machining Parameters	Level 001	Level 002	Level 003
V (m/min)	150	175	200
F (mm/edge)	0.15	0.20	0.25
D (mm)	1.00	1.20	1.40

**Table 5:** Resulted values using Taguchi L9 array.

Sl. No.	*MRR* (mm^3^/min)	*F_x_* (N)	*F_y_* (N)	*F_z_* (N)	*Ra* (µm)
1	5494.9095	−6.00	5.00	2.37	5.88
2	8165.0226	−4.25	6.50	5.40	4.92
3	12,038.8386	−6.90	2.74	4.75	4.58
4	10,396.7194	3.70	22.5	−13.00	5.06
5	10,063.401	−1.75	2.75	3.40	4.84
6	9147.9444	−10.50	10.90	3.70	4.72
7	12,051.4479	−5.00	4.50	2.19	5.56
8	11,318.707	−2.60	4.00	2.70	4.98
9	16,390.8962	−8.00	10.00	12.50	5.08

**Table 6:** Quality Loss ($Q_{ij}$).

Sl. No.	*MRR*	*F_x_*	*F_y_*	*F_z_*	*Ra*
1	3.3119 × 10^−8^	36	25	5.6169	34.5744
2	1.5 × 10^−8^	18.0625	42.25	29.16	24.2064
3	6.8997 × 10^−9^	47.61	7.5076	22.5625	20.9764
4	9.2514 × 10^−9^	13.69	506.25	169	25.6036
5	9.8744 × 10^−9^	3.0625	7.5625	11.56	23.4256
6	1.195 × 10^−8^	110.25	118.81	13.69	22.2784
7	6.8853 × 10^−9^	25	20.25	4.7961	30.9136
8	7.8056 × 10^−9^	6.76	16	7.29	24.8004
9	3.7222 × 10^−9^	64	100	156.25	25.8064

**Table 7:** Evaluation of S/N ratio ($S_{ij}$).

Sl. No.	*MRR*	*F_x_*	*F_y_*	*F_z_*	*Ra*
1	74.79921	−15.563	−13.9794	−7.49497	−15.3875
2	78.23915	−12.5678	−16.2583	−14.6479	−13.8393
3	81.61169	−16.777	−8.75501	−13.5339	−13.2173
4	80.33793	−11.364	−27.0437	−22.2789	−14.083
5	80.0549	−4.86076	−8.78665	−10.6296	−13.6969
6	79.22647	−20.4238	−20.7485	−11.364	−13.4788
7	81.62078	−13.9794	−13.0643	−6.80888	−14.9015
8	81.07594	−8.29947	−12.0412	−8.62728	−13.9446
9	84.29205	−18.0618	−20	−21.9382	−14.1173

**Table 8:** Scaled ratio of S/N.

Sl. No.	*MRR*	*F_x_*	*F_y_*	*F_z_*	*Ra*
1	1	0.313	0.715	0.956	0
2	0.66	0.505	0.59	0.494	0.714
3	0.327	0.235	1	0.566	1
4	0.453	0.583	0	0	0.601
5	0.481	1	0.998305	0.764	0.738
6	0	0	0.345	0.706	0.88
7	0.326	0.415	0.765	1	0.224
8	0.38	0.779	0.821	0.883	0.665
9	0.062	0.152	0.386	0.926	0.586

**Table 9:** Values of GRC and GRG.

Sl. No.	$\mathrm{GRC}(Y_{ij})$					*GRG_i_*
	*MRR*	*F_x_*	*F_y_*	*F_z_*	*Ra*	
1	0.333333	0.615006	0.411523	0.343407	1	0.540653742
2	0.431034	0.497512	0.458716	0.503018	0.411862	0.460428448
3	0.604595	0.680272	0.333333	0.469043	0.333333	0.48411537
4	0.524659	0.461681	1	1	0.454133	0.688094419
5	0.509684	0.333333	0.33371	0.39557	0.403877	0.395234919
6	1	1	0.591716	0.414594	0.362319	0.673725703
7	0.605327	0.546448	0.395257	0.333333	0.690608	0.51419459
8	0.568182	0.39093	0.378501	0.361533	0.429185	0.425666163
9	0.88968	0.766871	0.564334	0.350631	0.460405	0.606384252

**Table 10:** Level mean of GRG values.

Level	Speed (A)	Feed (B)	Depth of Cut (C)
1	0.4951	0.5810	0.5467
2	0.5857	0.4271	0.5850
3	0.5154	0.5881	0.4645
Delta	0.0906	0.1610	0.1610
Rank	3	1	2

**Table 11:** Analysis of Variance for GRG values.

Source	DF	Seq SS	Adj SS	Adj MS	F	P	% of Contribution
V	2	0.013564	0.013564	0.006782	7.23	0.122	15.45
F	2	0.049636	0.049636	0.024818	26.45	0.036	56.53
D	2	0.022726	0.022726	0.011363	12.11	0.076	25.88
Error	2	0.001877	0.001877	0.000938	--	--	2.13
Total	8	0.087804	--	--	--	--	100

## Data Availability

Not applicable.
